# Flow mediated vasodilation compared with carotid intima media thickness in the evaluation of early cardiovascular damage in menopausal women and the influence of biological and psychosocial factors

**DOI:** 10.1186/s12905-018-0648-3

**Published:** 2018-09-20

**Authors:** Mauricio Sanchez-Barajas, Lorena del Rocio Ibarra-Reynoso, Marco Antonio Ayala-Garcia, Juan Manuel Malacara

**Affiliations:** 1 Department of Internal Medicine, Instituto Mexicano del Seguro Social, General Hospital Zone/MF 21, Leon, Guanajuato Mexico; 2 Department of Medical Sciences, University of Guanajuato, León Campus, León, GTO Mexico; 3 Department of General Surgery, Instituto Mexicano del Seguro Social, General Hospital Subzone No 10, Guanajuato, Guanajuato Mexico

**Keywords:** Menopause, Cardiovascular risk, Flow-mediated vasodilation, Carotid intima media thickness, Heart rate variability, Submission

## Abstract

**Background:**

Women after menopause increase risk for cardiovascular disease and several factors may be related. The purpose was to study biological and psychosocial factors associated with early cardiovascular damage in pre- and postmenopausal women, assessed with carotid intima-media thickness vs flow-mediated dilatation.

**Methods:**

Women 45 to 57 years old were grouped in the pre- (*n* = 60), early (*n* = 58) and late post-menopause (*n* = 59). Anthropometric, metabolic and hormonal data were registered, as well as measures of depression, anxiety, submission, perceived stress, and sleep alterations. Heart Rate Variability was recorded to obtain the information regarding sympathovagal balance. Carotid intima-media thickness and flow-mediated dilatation were assessed by ultrasound. Two-way ANOVA and multiple regression model were used.

**Results:**

At late postmenopause, the carotid intima-media was thicker (*p* < 0.001) and flow-mediated dilatation decreased (*p* < 0.001). Carotid intima-media thickness was associated positively with age (p < 0.001), submission score (*p* = 0.029), follicle stimulating hormone levels (p < 0.001), and body mass index (*p* = 0.009). Flow-mediated dilatation was associated only with age (p < 0.001). Regarding heart rate variability, the time domain pNN50 measurement was higher in premenopausal women (*p* = 0.001), Low Frequency (LF) was higher in the two groups of postmenopausal (p = 0.001) and High Frequency (HF) higher in the early postmenopausal women (*p* = 0.042).

**Conclusions:**

Under our conditions carotid intima-media thickness had higher predictive value for early cardiovascular damage at menopause. The finding of the association of the submission score, indicates de influence of stress on vascular damage.

## Background

Menopause, the permanent cessation of menstruation results from ovarian senescence with depletion of ovarian follicles and decreased estrogen production [1]. Estrogenic deficiency during the menopausal transition frequently leads to somatic, psychological, and vasomotor symptoms, and sexual dysfunction. Women may suffer depression, anxiety and sleep alterations attributed to low estrogen levels with the interaction of psychosocial conditions [2].

Women, at this period of their life may have hot flashes, also associated with mood alterations that may be related to the influence of psychosocial conditions and the perception of their role in society [3]. Mood alterations like depression [4], and hostility [5] are predictors of mortality and cardiovascular events.

The incidence for cardiovascular diseases (CVDs) sharply increases after 50 years of age, making them the main cause of death in mature women, exceeding all cancer deaths [6].

Increased risk of CVDs, may be related to several physiological changes such as increased body fat [7], and an adverse lipid profile [8]. The vascular endothelium produces autoimmune, endocrine, and paracrine factors [9], of primary importance in vascular function. Endothelial dysfunction is a relevant event in atherogenesis and cardiovascular damage in postmenopausal women [10]. The SWAN study [11] reported that peri- and postmenopausal women had larger diameters of common carotid artery and adventitial than at premenopause, suggesting that estrogen diminution is associated with vascular damage.

Numerous factors alter the endothelial function, such as age [12], dyslipidemia, hypertension, obesity, smoking habit, diabetes mellitus, and psychosocial factors [13].

Carotid intima media thickness (C-IMT) is a widely used surrogate marker of subclinical atherosclerosis with established prognostic value [14]. Assessment of endothelial function using brachial artery flow-mediated dilatation (FMD) is another predictor of cardiovascular events [15], used for risk stratification [10].

Few studies have compared the efficacy of these two clinical measurements in the environment of menopause. Some authors indicate that at premenopause [16] and early menopause [17], the measurement of FMD is a more appropriate test for cardiovascular damage than C-IMT. However, a recent study supports C-IMT as a subclinical marker of vascular damage in postmenopause in the presence of metabolic syndrome [18].

The Heart Rate Variability (HRV) is an index of sympathovagal balance, which is altered in relation to mood disorders and cardiovascular risk [19]. HRV is negatively associated with C-IMT and its progression [20]. The sympathovagal tonus during the stress test is negatively correlated with C-IMT [20]. In a previous study, we found associated HRV and ultrasonographic carotid indices (resistive index, young’s elastic modulus, arterial compliance, arterial distensibility) in peri- and postmenopausal women [21], indicating a manifestation of autonomic imbalance and cardiovascular risk.

Considering the need for a reliable clinical evaluation of early cardiovascular risk, we studied biological and psychosocial factors associated with early cardiovascular damage in pre- and postmenopausal women, assessed with carotid intima-media thickness as a measure of subclinical atherosclerosis vs flow-mediated dilatation as a measure of endothelial function.

## Methods

### Study population

We carried out a descriptive, comparative study in 177 women invited at their home in urban and suburban areas of León, Mexico.

We included women hysterectomized, non-pregnant, non-lactating, without clinical evidence of acute or chronic, infectious, metabolic or cardiovascular disease. They did not have history of recent anxiolytics, antidepressants, analgesics, vitamins, or antibiotic consumption. They did not receive previously hormone replacement therapy.

We compared three groups according to menopausal stage, according STRAW criteria [22]. Women at pre-menopause, with regular menstrual periods (*n* = 60); at early postmenopause, women aged 45 to 57 and less than 5 years since the last menstrual (*n* = 58). Women older to 57 and with more than 5 years since the last menstrual period were classified at late postmenopause (*n* = 59).

This study was approved by the Committee of Bioethics of the University of Guanajuato. Eligible participants who accepted inclusion signed informed consent.

### Anthropometry data and body composition

We collected age, height, weight, and waist circumference. Body mass index (BMI) was calculated (kg/m^2^). Waist circumference was measured at the middle between the lower rib and the iliac crest.

### Data collection

We collected years of schooling, work as housewife or out of home, exercises at least 1 day/week (yes or no), smoking habit (yes or no), and alcohol consumption (at least one drink/week, yes or no), as described in our previous studies [20, 21]. Blood pressure was measured with a random zero sphygmomanometer in sitting position after a five minutes rest.

### Gyneco-obstetric history

The age at menarche, date of the last menstrual period, number of pregnancies, deliveries, abortions, cesarean sections and complications during pregnancy were also registered.

### Physical and psychological symptoms

Women answered a questionnaire on physical and psychological symptoms at menopause, as in our previous works [21, 23], to assess: hot flushes as sensations of transient heat in skin, sexual interest (yes or not), perceived stress, anxiety, depressive mood, sleep disturbances, submission and effort/reward imbalance.

We reported intensity of hot flushes as none = 0, slight = 1, moderate = 2, or severe = 3. Depressive mood and anxiety were evaluated with the Hamilton-Bech-Raphaelsen Scale [24]. Depressive mood was rated 0 to 4, with total scores ranging 0 to 26. The anxiety score is an 18-item self-reported questionnaire ranging from 0 to 18.

Perceived stress was measured using an instrument adapted from Cohen as us in previous reports [25, 26], with 14 questions and a score of 0 to 56.

For the effort-reward imbalance (ERI) evaluation, we used the instrument of Siegrist [27] adapted to the Spanish language [28]. This scale results from the ratio of extrinsic effort/reward, both factors collected with a Likert scale (1 to 5). Values above or below 1.0, indicate high or low risk, respectively. For submission score we used a modified Rathus Assertiveness Scaling [29], which contain 16 items, and a score ranging from 0 to 64. The evaluation of sleep was recorded in two items: difficulties sleeping and sleep disturbances, in three levels, with a total score of 0 to 6.

### Samples and laboratory procedures

#### Samples

Samples of peripheral blood were drawn after 12-h fasting. Serum was stored at − 80 °C until use.

#### Biochemical determinations

The concentrations of glucose, creatinine and lipid profile were determined by enzymatic colorimetric methods with precisions of 1.5%, 2.2% and 2.1% respectively.

#### Quantification of hormones

We quantified cortisol serum levels as biomarker of stress response. Follicle stimulating hormone (FSH) and serum morning cortisol were measured by ELISA commercial kits (ALPCO, USA), with respective intra- and interassay coefficients of variation of 3.4% and 7.7% for FSH and 2.9% and 3.8% for cortisol.

### Heart rate variability

According to the consensus Task Force of the European Society of Cardiology and the American Society of Electrophysiology [30], two periods for recording have been validated, at 5 min and 24 h. Recording was carried out in a fasting condition after ten minutes rest. The RS800CX clock (Polar, Finland) was used from 8:00 a.m. to 12:00 p.m. Smoking or alcohol beverages were not allowed for one day before study. During the hours of recording women realized the normal activities. The use of this instrument was validated as a convenient and inexpensive device for a reliable assessment of HRV (18).

The Time Domains studied were: *SDNN* (*Standard deviation of NN intervals in milliseconds)141 ± 39*: reflecting the influence of the sympathetic system. *rMSSD* (Square root of the mean squared differences of adjacent intervals in milliseconds) 27 ± 12: indicates the vagal influence on HRV. *pNN50* (The proportion of adjacent normal RR intervals differing more than 50 ms) 2–5%: A decrease in its normal value indicates cardiovascular risk, with parasympathetic influence.

Frequency Domains determined were: *HF (High frequency) 0.04–0.15 Hz*: is known to reflect mainly the effect mediated parasympathetic vagal tone. *LF (Low frequency) 0.15 to 0.40 Hz*: evaluates both sympathetic and parasympathetic inputs and has been associated to the role of baroreceptors. *LF/HF index1.5–2%*: is considered as an indicator of the sympathetic-parasympathetic imbalance.

*Power spectral measurements* were calculated according to the autoregressive model with an order size of 16. The spectral power of each frequency is expressed in absolute values ​​ (ms2/Hz), with logarithmic transformation. Statistical analysis was carried out using the MATLAB ® (The Math Works USA) [31] software, based on Fourier transformation [32–34].

### Assessment of early cardiovascular damage

The early cardiovascular damage was assessed with carotid intima-media thickness as a measure of subclinical atherosclerosis vs flow-mediated dilatation as a measure of endothelial function. For both tests, we used continuous variables, considering that provide more information than the use of cut-off points.

The assessment of endothelial function on the brachial artery flow-mediated dilatation was made with a high-resolution ultrasound Doppler according to the recommended guidelines of the American College of Cardiologists [35]. The test was carried out in our facilities at 8:00 am in a quiet, temperature-controlled room. In order to minimize the impact of short term hormonal changes, those measurements were made in the first seven days of the menstrual cycle in premenopausal women. Participants were asked to sleep a minimum of six hours, and not to consume caffeine beverages. The brachial artery flow-mediated dilatation was assessed by external B-mode ultrasound imaging 2–3 cm above the elbow (Acuson, 7.0 MHz linear transducer; Mountain View, CA, USA) according to the recommendations of the International Brachial Artery Task Force [35, 36]. Depths and gain settings were optimized to identify the lumen to vessel wall interface. The subject rested in the supine position for at least 30 min before the first scan and remained supine during the evaluation. Blood flow increase was induced by inflation of a pneumatic cuff placed around the forearm to a pressure at least 50 mmHg above systolic blood pressure. Five minutes later the cuff was rapidly deflated. The artery was scanned continuously for 90 s and recorded on a super-video home system tape for posterior analysis. The diameter of the brachial artery proximal to the elbow was manually measured at the peak of the R-wave at baseline and at 30, 60 and 90 s following cuff deflation just proximal to the elbow. FMD was defined as the maximal brachial artery diameter recorded between 30 and 90 s following cuff release minus the diameter at rest and divided by the diameter at rest (% FMD = (post-ischemia diameter - basal diameter) / basal diameter × 100) [35].

For the assessment of subclinical atherosclerosis, the carotid intima-media thickness was measured with 8.0 MHz linear transducer, in accordance to the consensus of the American Society of Echocardiography [37]. The average C-IMT was measured in the far wall of the distal third of both primitive carotid arteries. The C-IMT was defined as the segment between the edge blood-intima and media adventitia. The patient in supine or semi-supine position with the head in slight hyperextension and 45° contralateral rotation was studied as recommended by the consensus of Mannheim in 2007 [38].The image is centered on the back wall of each common carotid artery, a 1 cm segment proximal to the carotid bifurcation of each side.

Only the intimate (echogenic line) and the media (hypoechoic line) are included in the measurement, based on the manual movement of a cursor at three different points. The average of the three values was used for analysis [38]. Both the C-IMT and FMD measurements were performed by an expert in ultrasonography with a precision of 1.9% and 1.4% respectively.

### Statistical analyses

The data are reported as mean and standard deviation or percentage, as appropriate. Normality was assessed using the Kolmogorov-Smirnov test. Data from premenopausal and early and late postmenopausal women were compared using two-way ANOVA for independent variables. We carried out a multiple regression models taking as dependent variables C-IMT and FMD and as regressor candidates: age, BMI, mean arterial pressure, FSH, cortisol, high density lipoprotein (HDL-Cholesterol), Non HDL-Cholesterol, LF, HF, as well as the depression, anxiety, stress and submission scores. We carried out one model including all candidate regressors; considering the strong influence of age, we repeated the procedure excluding age. Then we did the of Bonferroni correction for multiple comparisons.

## Results

The general characteristic of women at premenopause, early and -late postmenopause are shown in Table 1. Women at premenopause had higher schooling, lower parity and marginally lower BMI; waist circumference and mean arterial pressure were not different.

**Table 1:** Comparison of characteristics of premenopausal and early and late postmenopausal women

	Premenopause women (n = 60)	Early postmenopause (*n* = 58)	Late postmenopause (*n* = 59)	*p*
Age(years)	41.4 ± 5.9	52.5 ± 4.1	57.7 ± 4.7	< 0.001^a, b, c^
Schooling(years)	11.08 ± 3.7	7.3 ± 4.6	8.08 ± 4.6	< 0.001^a, b^
Parity(number)	2.4 ± 1.8	4.3 ± 2.06	3.8 ± 2.2	< 0.001^a,b^
Body Mass Index(kg/m^2^)	27.3 ± 4.3	29.3 ± 5.1	28.7 ± 4.3	0.061
Waist circumference(cm)	91.1 ± 10.5	93.6 ± 11.8	91.1 ± 11.1	0.402
Mean arterial pressure(mmHg)	82.16 ± 5.30	84.13 ± 5.38	82.99 ± 5.89	0.151
Loss of sexual interest(range 0–3)	0.73 ± 0.7	1.62 ± 1.1	1.77 ± 1.1	< 0.001^a,b,c^
Intensity of hot flushes(1–3)	0.23 ± 0.4	0.86 ± 0.3	0.74 ± 0.4	< 0.001^a,b^
Perceived stress(range 0–64)	24.4 ± 5.8	22.4 ± 5.3	25.05 ± 6.3	0.043^c^
Anxiety(range 0–18)	4.7 ± 3.03	6.1 ± 3.8	5.7 ± 3.6	0.062
Depressive mood(range 0–26)	5.05 ± 4.3	5.4 ± 5.1	5.3 ± 4.4	0.871
Disturbed sleep(range 0–3)	0.26 ± 0.4	0.43 ± 0.4	0.52 ± 0.5	0.012^a,b^
Submission(range 0–64)	18.4 ± 9.9	24.2 ± 11.6	22.9 ± 9.2	0.006^a,b^
Effort-reward imbalance	0.3 ± 0.1	0.4 ± 0.5	0.3 ± 0.1	0.411
Glucose(mg/dl)	87.5 ± 9.8	92.6 ± 10.2	93.05 ± 11.1	0.006^a,b^
Triglycerides(mg/dL)	122.3 ± 57.7	148.6 ± 60.1	155.8 ± 68.8	0.009^a,b^
HDL-Cholesterol(mg/dL)	47.7 ± 11.7	55.6 ± 12.01	59.6 ± 9.5	< 0.001^a,b^
Non HDL-Cholesterol(mg/dL)	103.6 ± 31.2	103.2 ± 27.8	126.1 ± 34.1	< 0.001^b,c^
Cortisol(μg/dL)	10.7 ± 4.8	12.4 ± 3.91	13.4 ± 5.8	0.181
FSH(IU/L)	12.1 ± 20.09	49.3 ± 24.7	53.4 ± 21.3	< 0.001^a,b^
SDNN(ms)	100.6 ± 80.2	108.9 ± 74.2	90.3 ± 44.03	0.362
rMSSD(ms)	28.6 ± 11.0	22.5 ± 8.5	27.2 ± 12.6	0.007^a,c^
pNN50(ms)	4.3 ± 6.2	1.6 ± 1.7	2.4 ± 2.5	0.001^a,b^
LF(Hz)	0.02 ± 0.01	0.04 ± 0.02	0.03 ± 0.01	0.001^a,b^
HF(Hz)	0.06 ± 0.02	0.09 ± 0.09	0.08 ± 0.06	0.042^a^
LF/HF(Hz)	0.5 ± 0.2	0.5 ± 0.2	0.5 ± 0.3	0.702
C-IMT(mm)	0.59 ± 0.1	0.71 ± 0.1	0.77 ± 0.1	< 0.001^a,b,c^
Flow-mediated dilatation(%)	15.4 ± 10.2	10.8 ± 6.7	9.9 ± 6.3	< 0.001^a, b^

Data are shown as mean ± SD; high-density lipoprotein (HDL); FSH (Follicle stimulating hormone); SDNN (Standard deviation of NN intervals); rMSSD (Square root of the mean squared differences of adjacent intervals in milliseconds); pNN50 (The proportion of adjacent normal RR intervals differing more than 50 ms); LF (low frequency); HF (high frequency); C-IMT (intima –media thickness)

a (Premenopause Vs Early postmenopause)

b (Premenopause Vs Late postmenopause)

c (Early vs late postmenopause)

In regards to symptoms, the loss of sexual interest increases with the progression of menopause (*p* < 0.001). The scores of intensity of hot flashes (*p* < 0.001), sleep disruption (*p* = 0.01) and submission (*p* = 0.006) are higher in postmenopausal women.

In the analysis of metabolic data, we found mean glucose level higher in postmenopausal women (p = 0.006). The triglyceride levels, and HDL-Cholesterol were higher at postmenopause (*p* = 0.009, and *p* < 0.001 respectively). Non HDL-Cholesterol was higher only in late postmenopause group (p < 0.001).

As expected FSH levels increased at postmenopause (p < 0.001), but cortisol had similar levels in the three groups.

The analysis of heart rate variability showed that the time domain pNN50 was higher in the group at premenopause compared with two groups at postmenopause (*p* = 0.001). For frequency domains, LF was higher in the two groups of postmenopausal women (p = 0.001) and HF higher at early postmenopause (*p* = 0.042). The rest of the variables were not different among the groups. C-IMT, increases progressively at early and late post-menopause (*p* < 0.001). We found that flow-mediated vasodilatation of the brachial artery had lower values in both groups of women at post-menopause (*p* < 0.001).

**Fig. 1:**
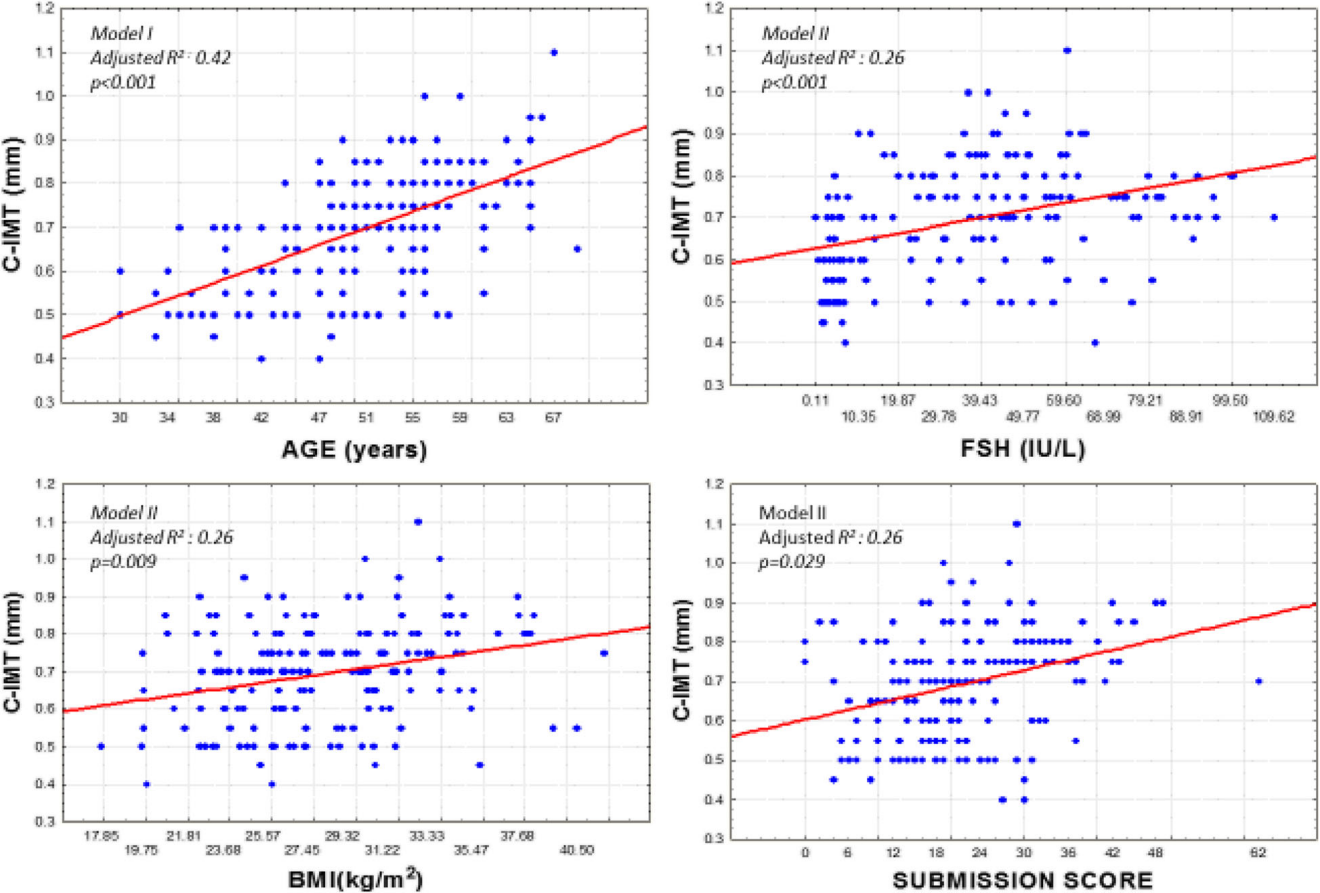
Association of C-IMT with age, FSH, BMI and submission score C-IMT (intima –media thickness); BMI (body mass index); FSH (Follicle stimulating hormone), *p* < 0.05.

A multiple regression model was constructed in order to define the factors associated of C-IMT and FMD testing factors representing age, obesity, estrogen deficiency, metabolic and hormonal variables, and depression, anxiety, stress, and submission scores, as well as heart rate variability. In the analysis of C-IMT we found that after Bonferroni correction, only age was positively associated (*p* < 0.001). Taking in consideration that variability of age also includes other factors that change with time, in a second model we removed age, and found FSH, BMI and submission scores positively associated to C-IMT (*p* < 0.001, *p* = 0.009 and *p* = 0.029). (Table 2 and Fig. 1).

**Table 2:** Factors associated with intima-media thickness

Dependent Variable: C-IMT *MODEL I (Adjusted R*^*2*^: *0.42)*	Beta±SE	t	*p*	*p* after Bonferroni correction
Age	0.55 ± 0.05	9.31	< 0.001	< 0.001
Submission score	0.15 ± 0.06	2.49	0.013	0.131
Body mass index	0.12 ± 0.06	2.04	0.042	0.421
MODEL II* *(Adjusted R*^*2*^: *0.26)* Excluding age from candidate regressors				
FSH	0.31 ± 0.06	4.73	< 0.001	< 0.001
Body mass index	0.22 ± 0.06	3.32	< 0.001	0.009
Submission score	0.20 ± 0.06	2.98	0.003	0.029
Non HDL-Cholesterol	0.18 ± 0.06	2.75	0.006	0.059

*SE* (standard error); *C-IMT* (intima –media thickness; Non high-density-lipoprotein-cholesterol (Non HDL- Cholesterol); *FSH* (Follicle stimulating hormone), p < 0.05

**Fig. 2:**
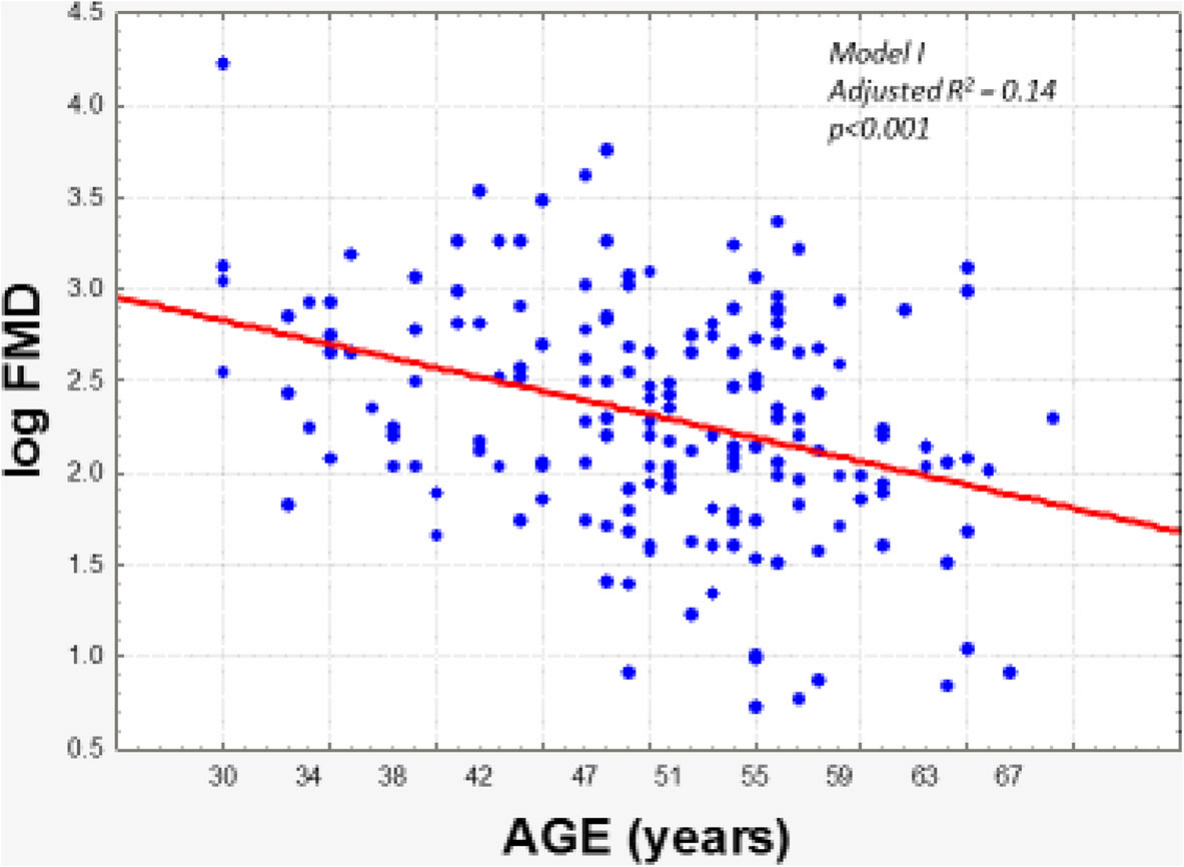
Association of the Flow-mediated dilatation with age FMD (flow-mediated dilatation), p < 0.05.

Next, we analyzed the factors associated with FMD. Considering a departure from normality of the variable we carried out analysis after log transformation and found that only age was associated (negatively, *p* < 0.001), (Table 3 and Fig. 2). In a second model removing age, no variable was significant.

**Table 3:** Factors associated with Flow-mediated dilatation

Dependent variable: Log FMD MODEL I *(Adjusted R*^*2*^ *= 0.14)*	Beta±S.E.	t	*p*	*P* after Bonferroni correction
Age	−0.30 ± 0.07	- 4.21	< 0.001	< 0.001
Submission score	−0.19 ± 0.07	- 2.75	0.006	0.078
MODEL II* *(Adjusted R*^*2*^: *0.12)* Excluding age from candidate regressors				
Submission score	−0.19 ± 0.07	- 2.69	0.008	0.069
HDL-Cholesterol	−0.18 ± 0.07	- 2.59	0.010	0.093
Non HDL-Cholesterol	−0.16 ± 0.07	- 2.35	0.019	0.171
Body mass index	−0.17 ± 0.07	- 2.29	0.023	0.207

*SE* (standard error); *FMD* (Flow-mediated dilatation); Non high-density-lipoprotein-cholesterol (Non HDL- Cholesterol), high-density-lipoprotein-cholesterol (HDL-Cholesterol), p < 0.05

The time since menopause was tested in all the multiple regression models, however the inclusion of this factor did not modify the final model.

**Fig. 3:**
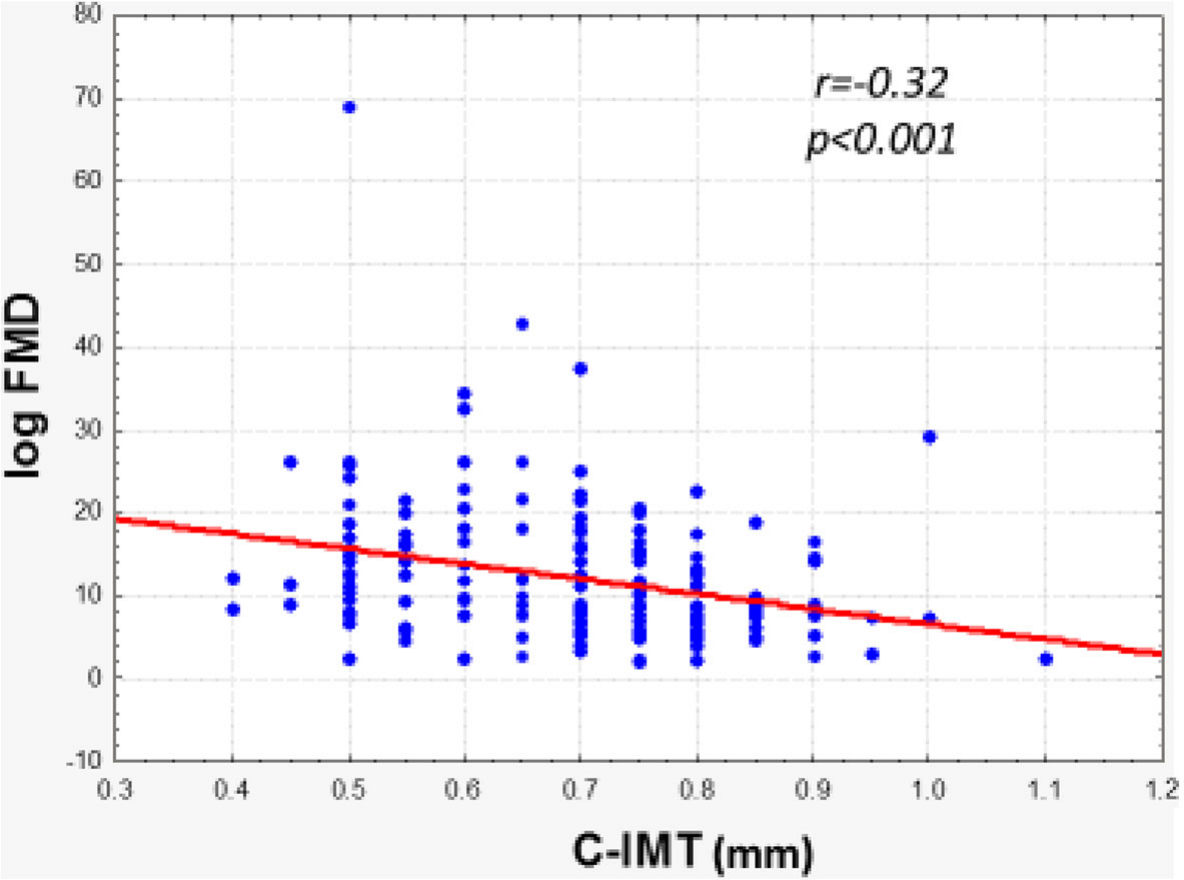
Association of the Flow-mediated dilatation with intima-media thickness FMD (flow-mediated dilatation); C-IMT (intima –media thickness), p < 0.05.

Finally, we analyzed the association between FMD and C-IMT, and found them strongly associated (p < 0.001) as shown in Fig. 3.

## Discussion

Women after menopause undergo increased risk for atherosclerotic coronary artery disease [39, 40]. This process has been attributed to the decrease of endogenous estrogen on the cardiovascular system. The progression of the effects of estrogen deficiency is indicated by the increase of FSH. The endothelium plays an important role in cardiovascular protection, the vascular tone, as well as in coagulation and inflammatory response [41].

In this work, we found at post-menopause adverse metabolic conditions: higher triglyceride, glucose and non-HDL-cholesterol. However, HDL-cholesterol increased, a factor considered favorable against vascular damage. We found also at post-menopause increased scores related to mood and psychosocial indicators such as disturbed sleep and submission, related to stressful conditions. In regards to HRV, higher time domain pNN50 and lower LF were found at pre-menopause indicating higher sympathetic tone. These findings are representative of conditions that regularly occur in women at these stages of life and agree with previous information [42].

In our study the progressive increase of C-IMT, and decrease in FMD, indicate vascular damage. We are not aware of studies comparing the values of FMD and C-IMT in the three stages of menopause. The high correlation of results between these studies supports their value for evaluation of early cardiovascular damage.

In this work, the stronger factor associated with both indicators of early cardiovascular damage was age. However, the correlation was higher for C-IMT than for Log(FMD), with R^2^ values of 0.42 vs 0.14 respectively. We interpreted this to mean that C-IMT is a better predictor of early cardiovascular damage. We considered that the variance explained by age includes the variance related to the influence of diverse factors related to vascular deterioration. Therefore, in order to disclose the effect of other factors, we repeated the multiple regression models excluding age as candidate regressor. Model II for C-IMT showed the significant association of FSH, BMI, and the submission score. Being FSH an indicator of estrogen diminution, we interpreted these associations to indicate that higher FSH values reflecting the diminished effect of the extensively discussed protective effect on estrogens on vascular damage [43]. Estrogen deficiency induces not only atherogenic lipid abnormalities, but also permit procoagulant oxidative mechanisms and endothelial dysfunction [44]. Previous studies show that estrogen deficiency in premenopausal women with amenorrhea, leads to decreased FMD [45]. The postmenopausal status is associated with higher C-IMT, and with an unstable plaque. It is still unclear whether different menopause stages have an effect on C-IMT differentially. Bechlioulis et al. [17] showed no differences in the C-IMT between early menopause and perimenopause. In our study the C-IMT increases from pre-menopause to early and late post-menopause, which may indicate the continued effect of estrogen deprivation and other damaging factors on the endothelium [46].

Previous studies have also shown correlations between BMI and increased C-IMT [47–49]. Obesity is an important factor for carotid atherosclerosis [50]. In addition to BMI, altered body-fat distribution and ectopic fat deposition are strongly associated with mortality and morbidity attributable to CVDs.

The influence of stress and psychosocial factors on cardiovascular damage has been proposed since long time. A study with female monkeys reported that the subordinate social status correlated with coronary artery atherosclerosis [51], however it has been difficult to prove in humans. Our group have studied various indicators of stress such as the perception of stress, depression, anxiety, dominance and submission dimensions, as well as cortisol measurement. In this work, the submission score was significantly associated with C-IMT. In a previous work, we reported the submission score positively associated with depression, perceived stress, anxiety and hot flashes in post-menopausal women (3). We consider that this is an interesting finding because in modern and traditional societies mature women are labile to the stress induced by insufficient opportunities for a satisfactory role in society. Another data of psychosocial interaction with biological influences, is the correlation of dominance scores with the estrogen receptor polymorphisms [3].

A recent research on FMD and C-IMT in postmenopausal women, reported that C-IMT was associated with metabolic syndrome at this stage [51]. The association of non-HDL-Cholesterol with C-IMT is congruent with the effect of dyslipidemia on vascular damage, as a permissive of atherosclerosis, and stimulation of monocyte infiltration and migration [18]. The increase in HDL-Cholesterol at post-menopause is contrary to previous information [52], but consistent with other reports [53].

The FMD and C-IMT are two inverse indicators of subclinical cardiovascular disease, used for cardiovascular risk stratification. In our conditions, C-IMT was associated with more factors, in a model with higher correlations coefficients, which we interpreted to support a higher predictive value.

A limitation of our study is its cross-section design in which association is deduced only on statistical basis without temporal precedence. Another limitation for more defined effects is that we did not measure biochemical indicators of endothelial dysfunction.

## Conclusion

In conclusion, in menopause the early of cardiovascular damage, can be assessed mainly by C-IMT measurement. The higher submission scores associated with increased C-IMT may be an interesting point about socioeconomic conditions that deserve further investigation of early cardiovascular damage in women during menopause.

## Abbreviations

BMI: Body mass index
C-IMT: Carotid intima media thickness
CVDs: Cardiovascular diseases
ERI: Effort-reward imbalance
FMD: Flow-mediated dilatation
FSH: Follicle stimulating hormone
HDL-Cholesterol: High density lipoprotein
HF: High frequency
HRV: The heart Rate Variability
LF: Low frequency
pNN50: The proportion of adjacent normal RR intervals differing more than 50 ms
rMSSD: Square root of the mean squared differences of adjacent intervals
SDNN: Standard deviation of NN intervals.
